# Integration of tumor inflammation, cell proliferation, and traditional biomarkers improves prediction of immunotherapy resistance and response

**DOI:** 10.1186/s40364-021-00308-6

**Published:** 2021-07-07

**Authors:** Sarabjot Pabla, R. J. Seager, Erik Van Roey, Shuang Gao, Carrie Hoefer, Mary K. Nesline, Paul DePietro, Blake Burgher, Jonathan Andreas, Vincent Giamo, Yirong Wang, Felicia L. Lenzo, Margot Schoenborn, Shengle Zhang, Roger Klein, Sean T. Glenn, Jeffrey M. Conroy

**Affiliations:** 1 OmniSeq, Inc, 700 Ellicott Street, Buffalo, NY 14203 USA; 2 Roswell Park Comprehensive Cancer Center, Elm and Carlton Streets, Buffalo, NY 14206 USA

**Keywords:** Inflammation, Cell proliferation, Pembrolizumab, Nivolumab, Ipilimumab, Algorithmic analysis, Inflamed, Borderline, Non-inflamed

## Abstract

**Background:**

Contemporary to the rapidly evolving landscape of cancer immunotherapy is the equally changing understanding of immune tumor microenvironments (TMEs) which is crucial to the success of these therapies. Their reliance on a robust host immune response necessitates clinical grade measurements of immune TMEs at diagnosis. In this study, we describe a stable tumor immunogenic profile describing immune TMEs in multiple tumor types with ability to predict clinical benefit from immune checkpoint inhibitors (ICIs).

**Methods:**

A tumor immunogenic signature (TIGS) was derived from targeted RNA-sequencing (RNA-seq) and gene expression analysis of 1323 clinical solid tumor cases spanning 35 histologies using unsupervised analysis. TIGS correlation with ICI response and survival was assessed in a retrospective cohort of NSCLC, melanoma and RCC tumor blocks, alone and combined with TMB, PD-L1 IHC and cell proliferation biomarkers.

**Results:**

Unsupervised clustering of RNA-seq profiles uncovered a 161 gene signature where T cell and B cell activation, IFNg, chemokine, cytokine and interleukin pathways are over-represented. Mean expression of these genes produced three distinct TIGS score categories: strong (*n* = 384/1323; 29.02%), moderate (*n* = 354/1323; 26.76%), and weak (*n* = 585/1323; 44.22%). Strong TIGS tumors presented an improved ICI response rate of 37% (30/81); with highest response rate advantage occurring in NSCLC (ORR = 36.6%; 16/44; *p* = 0.051). Similarly, overall survival for strong TIGS tumors trended upward (median = 25 months; *p* = 0.19). Integrating the TIGS score categories with neoplastic influence quantified via cell proliferation showed highly proliferative and strong TIGS tumors correlate with significantly higher ICI ORR than poorly proliferative and weak TIGS tumors [14.28%; *p* = 0.0006]. Importantly, we noted that strong TIGS and highly [median = not achieved; *p* = 0.025] or moderately [median = 16.2 months; p = 0.025] proliferative tumors had significantly better survival compared to weak TIGS, highly proliferative tumors [median = 7.03 months]. Importantly, TIGS discriminates subpopulations of potential ICI responders that were considered negative for response by TMB and PD-L1.

**Conclusions:**

TIGS is a comprehensive and informative measurement of immune TME that effectively characterizes host immune response to ICIs in multiple tumors. The results indicate that when combined with PD-L1, TMB and cell proliferation, TIGS provides greater context of both immune and neoplastic influences on the TME for implementation into clinical practice.

**Supplementary Information:**

The online version contains supplementary material available at 10.1186/s40364-021-00308-6.

## Background

Since the approval of the first immune checkpoint inhibitor (ICI) for melanoma in 2011, the landscape of cancer therapies has changed dramatically, combining biological response with genomics knowledge to change treatment paradigms and improve clinical outcomes [1]. Immunotherapies have shown to significantly improve clinical endpoints such as progression free survival (PFS) and overall survival (OS) in multiple cancer subtypes compared to chemotherapy alone [2]. Despite the tremendous efficacy of ICIs in some patients, other patients fail to respond to therapy, while others can develop severe autoimmune toxicity [1, 3]. To maximize treatment benefit and develop personalized therapeutic strategies, genomic and immune biomarkers, such as PD-L1 and tumor mutational burden (TMB), are utilized to guide therapeutic decisions based on tumor subtype [2, 3]. Although biomarker analyses regularly guide treatment decisions in standard of care clinical settings, single biomarkers alone are insufficient to adequately predict therapeutic response in some patients [2]. As a result, there is increased demand for the development of predictive assays which consider the multitude of networks and cellular phenotypes that complicate the immune tumor microenvironment (TME).

Proximity between tumor cells and immune cells is essential, though not entirely sufficient for immunotherapy efficacy, as tumors can avoid destruction by immune escape mechanisms such as downregulation of antigens, recruitment of immune suppressors, and upregulation of receptors that downregulate tumor-infiltrating lymphocytes (TILs) [4, 5]. It is well known that the success of ICIs depends upon the mobilization of the immune system within the TME where cancer cells interact with stromal cells [5, 6]. Therefore, the development of a biomarker detection modality inclusive of both cell proliferation and inflammation biomarkers is necessary to improve patient management.

A recent study by *Ayers* et al. analyzed RNA-seq gene expression profile (GEP) consisting of IFN-gamma genes, chemokine expression, cytotoxic activity and immune resistance genes along with PD-L1 and TMB. While the T cell-inflamed GEP signatures correlated with clinical benefit, the addition of all the gene profiles in the GEP was not always of sufficient sensitivity for the clinical benefit [7, 8]. Here we describe a multivariate approach to investigate combinations of immune and neoplastic influences responsible for response to ICI beyond a comprehensive immunogenic signature.

## Methods

### Patients and clinical data

Fifteen collaborating institutions obtained approval by their respective institutional review boards (IRBs) to submit existing de-identified specimens and associated clinical data for use in this study. This study involves two separate cohorts, namely, a discovery cohort of clinically tested solid tumors used for development of the immunogenic signature and a retrospective cohort for which response to ICI therapy and overall survival was available. For the discovery cohort, a total of 1323 patients were included [Supplementary Tables S1, S10], based on the following criteria, (1) Availability of high-quality gene expression data from samples clinically tested by a CLIA approved targeted RNA-seq assay [9]; (2) Samples that pass clinically approved tissue, nucleic acid and sequencing QC metrics; (3) Samples that have less than 50% necrosis and at least 5% tumor purity; and (4) Availability of other primary immune biomarkers such as PD-L1 IHC (TPS %) and TMB.

The retrospective cohort of 242 tumors were from patients treated with ICIs including non-small cell lung cancer (NSCLC) (*n* = 110) [10], melanoma (*n* = 78) [11], and renal cell carcinoma (RCC) cases (*n* = 54) [Table 1, Supplementary Table S11] [12]. Inclusion criteria comprised of treatment by an FDA approved ICI agent as of November 2017, had follow up and survival from first ICI dose and evaluable response based on RECIST v1.1. RECIST responses of complete response (CR) and partial response (PR) were classified as responders, whereas, stable disease (SD) or progressive disease (PD) were classified as non-responders. Duration of response was not available for all patients and not included for final analysis.

**Table 1:** Clinical characteristics of the retrospective cohort

	NSCLC Patients	Melanoma			RCC
	Patients (n = 110)	All Case (***n*** = 78)	Pre-ipi approval (***n*** = 4)	Post-ipi approval (***n*** = 74)	ICI Treated (***n*** = 54)
**Age at initial diagnosis (years)**					
**< 30**	**1 (0.9%)**				
**30–39**		**7 (9.0%)**	**1 (25.0%)**	**6 (8.1%)**	**1 (1.9%)**
**40–49**	**3 (2.7%)**	**14 (17.9%)**	**1 (25.0%)**	**13 (17.6%)**	**6 (11.1%)**
**50–59**	**26 (23.6%)**	**13 (16.7%)**	**1 (25.0%)**	**12 (16.2%)**	**21 (38.9%)**
**60–69**	**41 (37.3%)**	**19 (24.4%)**	**1 (25.0%)**	**18 (24.3%)**	**16 (29.6%)**
**70–79**	**30 (27.3%)**	**18 (23.1%)**		**18 (24.3%)**	**10 (18.5%)**
**≥ 80**	**9 (8.2%)**	**7 (9.0%)**		**7 (9.5%)**	
**Mean**	**65.4**	**60.6**	**48**	**61.3**	**59.5**
**Year of diagnosis (Range)**	**2007–2017**	**1990–2016**	**2004–2009**	**1990–2016**	**1981–2016**
**Sex**					
**Female**	**58 (52.7%)**	**26 (33.3%)**	**2 (50.0%)**	**24 (32.4%)**	**14 (25.9%)**
**Male**	**52 (47.3%)**	**52 (66.7%)**	**2 (50.0%)**	**50 (67.6%)**	**40 (74.1%)**
**Race**					
**White**	**91 (82.7%)**	**78 (100.0%)**	**4 (100.0%)**	**74 (100.0%)**	**41 (75.9%)**
**Other**	**14 (12.7%)**				**7 (13.0%)**
**Unknown**	**5 (4.5%)**				**6 (11.1%)**
**Vital status at last follow up**					
**Alive**	**55.00 (50.0%)**	**46.00 (59.0%)**	**2.00 (50.0%)**	**44.00 (59.5%)**	**31.00 (57.4%)**
**Dead**	**55.00 (50.0%)**	**32.00 (41.0%)**	**2.00 (50.0%)**	**30.00 (40.5%)**	**23.00 (42.6%)**
**Checkpoint inhibitor**					
**atezolizumab**	**2 (1.8%)**				
**ipilimumab**		**35 (44.9%)**	**3 (75.0%)**	**32 (43.2%)**	
**ipilimumab + nivolumab**	**2 (1.8%)**	**10 (12.8%)**	**1 (25.0%)**	**9 (12.2%)**	
**nivolumab**	**71 (64.5%)**	**2 (2.6%)**		**2 (2.7%)**	**54 (100.0%)**
**pembrolizumab**	**35 (31.8%)**	**31 (39.7%)**		**31 (41.9%)**	
**Months of follow up**					
**< 1**	**48 (43.6%)**				**21 (38.9%)**
**3**	**6 (5.5%)**	**1 (1.3%)**		**1 (1.4%)**	**1 (1.9%)**
**6**	**17 (15.5%)**	**12 (15.4%)**		**12 (16.2%)**	**5 (9.3%)**
**10**	**22 (20.0%)**	**15 (19.2%)**		**15 (20.3%)**	**14 (25.9%)**
**> 10**	**17 (15.5%)**	**50 (64.1%)**	**4 (100.0%)**	**46 (62.2%)**	**13 (24.1%)**
**Median**	**8**	**12.5**	**63**	**12**	**10**

### Quality assessment of clinical FFPE tissue specimens

Tissue sections from FFPE blocks were cut at 5 μm onto positively charged slides. One cut section from each tissue sample was stained with H&E and assessed by a board-certified anatomical pathologist for adequacy of tumor representation, the quality of tissue preservation, evidence of necrosis, or issues with fixation or handling were present. Specimens containing < 5% tumor tissue and > 50% necrosis were excluded from analysis. In general, tissue from 3 to 5 unstained slide sections, with or without tumor macrodissection, was required to achieve the assay requirements for RNA (10 ng) and DNA (20 ng) input.

### Immunohistochemical studies

The expression of PD-L1 on the surface of cancer cells was assessed in all cases regardless of tumor type by means of the Dako PD-L1 IHC 22C3 pharmDx (Agilent, Santa Clara, CA). PD-L1 levels were scored by a board-certified anatomic pathologist as per published guidelines [13], with a tumor proportion score (TPS) ≥ 1% considered as positive result (PD-L1+). PD-L1 TPS < 1% was considered negative (PD-L1-).

Tissue sections were also examined for CD8 T-cell infiltration using anti-CD8 antibodies (C8/144B; Agilent, Santa Clara, CA) and classified into non-infiltrating, infiltrating, or excluded CD8 infiltration groups. Cases where a sparse number of CD8+ T-cells infiltrated clusters of neoplastic cells with less than 5% of the tumor showing an infiltrating pattern were designated non-infiltrating, while those showing frequent infiltration of neoplastic cell clusters in an overlapping fashion, at least focally, in more than 5% of the tumor were designated infiltrating. Cases where more than 95% of CD8+ T-cells were restricted to the tumor periphery or interstitial stromal areas and did not actively invade clusters of neoplastic cells were designated as excluded.

### Nucleic acid isolation, gene expression and TMB

DNA and RNA were co-extracted from each sample and processed for gene expression by RNA-seq and TMB by DNA-seq, as previously described [9]. Nucleic acids were quantitated by Qubit fluorometer (Thermo Fisher Scientific) using ribogreen staining for RNA and picogreen staining for DNA. Gene expression were evaluated by RNA sequencing of 395 transcripts on samples that met validated quality control (QC) thresholds [9]. TMB was measured by DNA sequencing of the full coding region of 409 cancer related genes as non-synonymous mutations per megabase (Mut/Mb) of sequenced DNA on samples with > 30% tumor nuclei. RNA and DNA libraries were sequenced to appropriate depth on the Ion Torrent S5XL sequencer (Thermo Fisher Scientific).

### Data analyses

Using the Torrent Suite plugin immuneResponseRNA (Thermo Fisher Scientific), RNA-seq absolute reads were generated for each transcript [9]. In each case, absolute read counts from the NTC were used as the library preparation background which was subtracted from the absolute read counts of the same transcript in all other samples of the same batch. To facilitate the comparability of NGS measurements across runs for evaluation and interpretation, background-subtracted read counts were normalized into normalized reads per million (nRPM) values by comparing each housekeeping (HK) gene background-subtracted read against an already-determined HK RPM profile. For each gene, nRPM expression values are converted to percentile rank of 0–100 when compared to a reference population of 735 solid tumors of 35 histologies [9] [Supplementary Fig. S1].

Initial visualization of the overall gene expression landscape of the discovery cohort was performed on the gene expression rank values using unsupervised hierarchical clustering with Pearson’s correlation (R) used as a measure of distance (phase 1). These results were then refined using k-means (k = 3) clustering to generate three stable clusters of patients (phase 2). Panther pathway enrichment analysis of these gene clusters distinguished them as cancer testis antigen genes, genes associated with the inflammation response, and other immune and neoplasm genes [Supplementary Tables S2, S3]. The 161-gene cluster associated with the inflammation response was termed the tumor immunogenic signature (TIGS), as the expression of these genes closely followed the degree of inflammation presented by each of the three patient clusters (phase 3) [Supplementary Fig. S2].

For each patient, a TIGS was calculated as mean expression rank of these 161 transcripts (phase 4). To derive clinically meaningful cutoffs for TIGS, we calculated overall average and standard deviation of TIGS across the three patients cluster of inflamed, borderline, and non-inflamed tumors [Supplementary Table S5]. Cutoff for strong immunogenicity (IS = 62) was derived as [Median TIGS]_Borderline_ + 2 × [Std. Dev. TIGS]_Borderline_, and similarly, for weak immunogenicity (IS = 43) was derived as, [Median TIGS]_Noninflamed_ + 2 × [Std. Dev. TIGS]_Noninflamed_, where TIGS = immunogenicity score. Any TIGS score between 62 and 43 was classified as moderate immunogenicity. For retrospective cohort with clinical outcome and survival data, we performed survival analyses using a log-rank test on 5-year Kaplan-Meier survival curves. We used a Cox proportional hazards model to calculate hazard ratios (HRs) and 95% confidence intervals [CIs] and *p* values for testing the effect of co-variates (tumor type, age, gender, PD-L1 IHC status, and TMB status) on overall survival. Comparison of ICI response rates was performed using Chi-square test with Yate’s continuity correction to test for significant differences in ICI response for various biomarker groups. [Supplementary Fig. S2].

## Results

### Tumor immunogenic signature (TIGS)

**Fig. 1:**
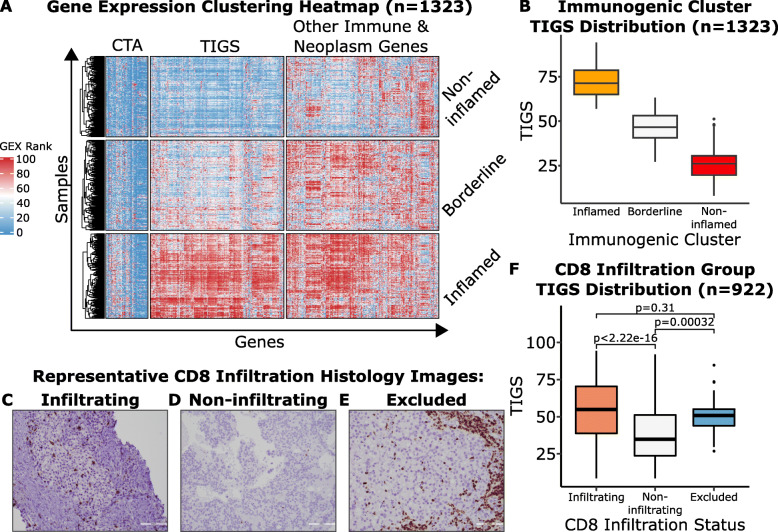
Discovery cohort gene expression clusters (**A**), and association with TIGS clusters (**B**), CD8 IHC patterns of T-cell infiltration (**C**–**E**), and TIGSdistribution within CD8 cohort (**F**). **A**. Unsupervised clustering of 1323 clinical RNA-seq profiles yield three immunogenic clusters, namely, inflamed (*n* = 439/1323; 33.18%), borderline (*n* = 467/1323; 35.30%) and non-inflamed (*n* = 417/1323; 31.52%). The tumor immunogenic signature (TIGS) cluster of genes contains 161-genes that are over-represented by T & B cell activation pathways along with IFNg, chemokine, cytokine and interleukin pathways. Mean expression of the 161 genes constituting the TIGS cluster produces the TIGS score. **B**. Distributions of the TIGS of the samples in each of the three sample clusters. **C**-**E**. Representative CD8 immunohistochemistry images of T cell infiltration patterns of Infiltrating (**C**), Non-infiltrating (**D**), and excluded (**E**). **F**. The distribution of immunogenic scores for tumors in the discovery cohort with strongly infiltrating, non-infiltrating, and excluded CD8 T cell infiltration patterns

Unsupervised hierarchical clustering of all genes sequenced in the discovery cohort revealed three clusters of coexpressing genes. Refining these results using k-means (k = 3) clustering generated three stable clusters of genes and three clusters of patients (non-inflamed, borderline, and inflamed) [Fig. 1a]. Pathway analysis of these gene clusters distinguished them as cancer testis antigen genes, genes associated with the inflammation response, and other immune and neoplasm genes [Supplementary Tables S2, S3]. The 161 genes associated with the inflammation response were termed the TIGS, as the expression of these genes closely followed the degree of inflammation presented by each of the three patient clusters [Fig. 1b]. The distributions of the immunogenic scores of the all samples in each of sample cluster were used to establish boundaries between the strong, moderate, and weak immunogenic score groups.

To assess agreement of the algorithmic TIGS with observed immune cell infiltration, we analyzed the distribution of immunogenic score within three major types of CD8 infiltration patterns estimated by IHC (infiltrating/strongly infiltrating, non-infiltrating, and excluded) [Fig. 1c-e]. As expected, the median immunogenic score of infiltrating/strongly infiltrating samples (*n* = 493) was 54.85, whereas the median immunogenic score of noninfiltrating samples (*n* = 403) was significantly lower (median = 34.84; *p* = 2.22E-16). Interestingly, excluded phenotype (*n* = 26) of immune infiltration had a median immunogenic score similar to the strongly/moderately infiltrating phenotype (median = 50.83; *p* = 0.31), but significantly higher than the noninfiltrating pattern (*p* = 0.00032) [Fig. 1f].

### TIGS and clinical outcomes

**Fig. 2:**
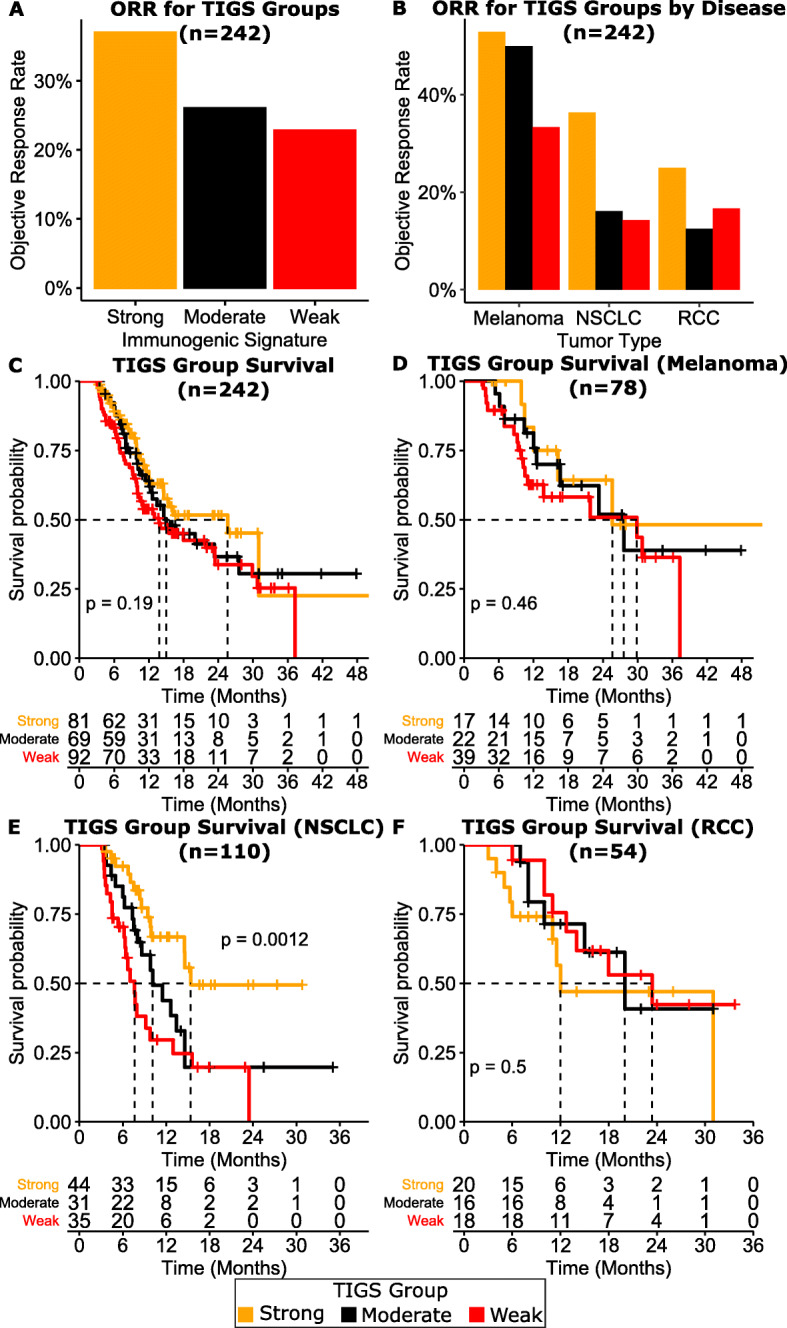
TIGS and ORR to ICI across all tumors in retrospective cohort (A) and within tumors (B). TIGS and survival across all tumors (C), melanoma (D), NSCLC (E), and RCC (F). **A.** Objective response rates (ORR) observed in the retrospective cohort for each TIGS group. **B.** ORR observed in each TIGS group for three disease types within the retrospective cohort. **C-F.** Survival curves for each TIGS group in the retrospective cohort **(C),** melanoma (**D**), NSCLC (**E),** RCC (**F**)

To assess clinical utility, TIGS was used to classify a previously published retrospective cohort of 242 samples with ICI outcomes (melanoma, NSCLC, and RCC) into strongly, moderately, and weakly immunogenic groups [11, 12, 14] [Fig. 2a]. Strongly immunogenic tumors showed higher objective response rate (ORR) compared to weakly immunogenic tumors (37% vs 23%; *p* = 0.06) to checkpoint inhibition in the retrospective cohort. Tumor type-specific analysis showed similar results in melanoma (53% vs. 33%; *p* = 0.27), NSCLC (36% vs. 14%; *p* = 0.05), and RCC (25% vs 16%; *p* = 0.8) [Fig. 2b] [Supplementary Table S4].

Next, we investigated the impact of immunogenic score on overall survival in the retrospective cohort. Even though there was no significant difference in overall survival of strongly inflamed compared to weakly inflamed tumors (*p* = 0.19), we observed a clear separation of median survival between the two groups (25.6 months vs. 13.8 months) [Fig. 2c]. Multivariate analysis using Cox proportional hazard model revealed that weakly inflamed TIGS category had a significantly high hazard ratio (HR = 1.83 [1.09–3.06]; *p* = 0.022) compared to strongly inflamed category [Supplementary Fig. S3]. We further investigated the source of this survival difference by performing tumor type-specific survival analysis, which showed that most of the survival difference can be attributed to NSCLC cases (*p* = 0.0012; 15.4 months vs. 7.63 months) [Fig. 2d-f, Supplementary Fig. S4-S6; Supplementary Table S5]. This NSCLC survival effect was supported by the multivariate Cox proportional hazard analysis where melanoma (HR = 0.39 [0.24–0.66]; *p* < 0.001) and RCC (HR = 0.44 [0.24–0.81]; *p* = 0.008) had significantly less effect on overall survival difference compared to NSCLC [Supplementary Fig. S3]. Age, gender and TMB status had no significant association to overall survival (*p* > 0.05) [Supplementary Fig. S3]. Interestingly, multivariate analysis of PD-L1 IHC status showed that negative cases showed trend towards worse survival (HR = 1.51[0.93–2.45]; *p* = 0.095) [Supplementary Fig. S3].

### TIGS and traditional biomarkers

**Fig. 3:**
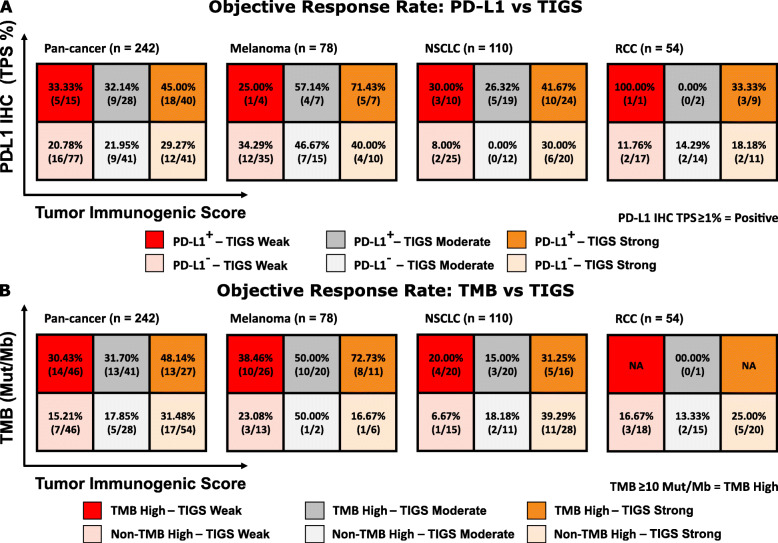
ORR to ICI in retrospective cohort combining TIGS with traditional biomarkers; PD-L1 (A) and TMB (B). **A.** ORR for each subgroup when TIGS is used in conjunction with PD-L1 status, by disease type. **B.** ORR for each subgroup when TIGS is used in conjunction with TMB status, separated by disease type

To further investigate the utility of TIGS, we studied the predictive capacity of TIGS in conjunction with traditional biomarkers for response to ICI therapy such as PD-L1 expression and TMB high. The combination of TIGS and PD-L1 shows an additive effect on objective response rate to ICI therapy in the retrospective cohort [Fig. 3a]. A similar effect was observed for TMB [Fig. 3b]. In general, PD-L1+, strongly immunogenic patients had the highest clinical response rate for all three cancer types (excluding single-sample groups), and PD-L1-, weakly immunogenic patients had the lowest response rate (or in the case of melanoma, the second-lowest). Interestingly, PD-L1 and TMB in combination did not show a similar effect [Supplementary Fig. S7]. In melanoma, TMB high, strongly inflamed patients had an ORR of 72.73%, while TMB low, strongly inflamed patients had a response rate of 16.67%.

Finally, combining TIGS with PD-L1 and TMB status for NSCLC, melanoma and RCC, the prediction of objective response becomes even more robust [Supplementary Fig. S7]. A significantly higher [*p* = 0.0001] objective response rate of 69.23% was observed for PD-L1 positive, TMB high, strongly inflamed tumors, compared to an objective response rate of only 10.53% for PD-L1 negative, non-TMB high, weakly inflamed tumors.

### TIGS and cell proliferation

**Fig. 4:**
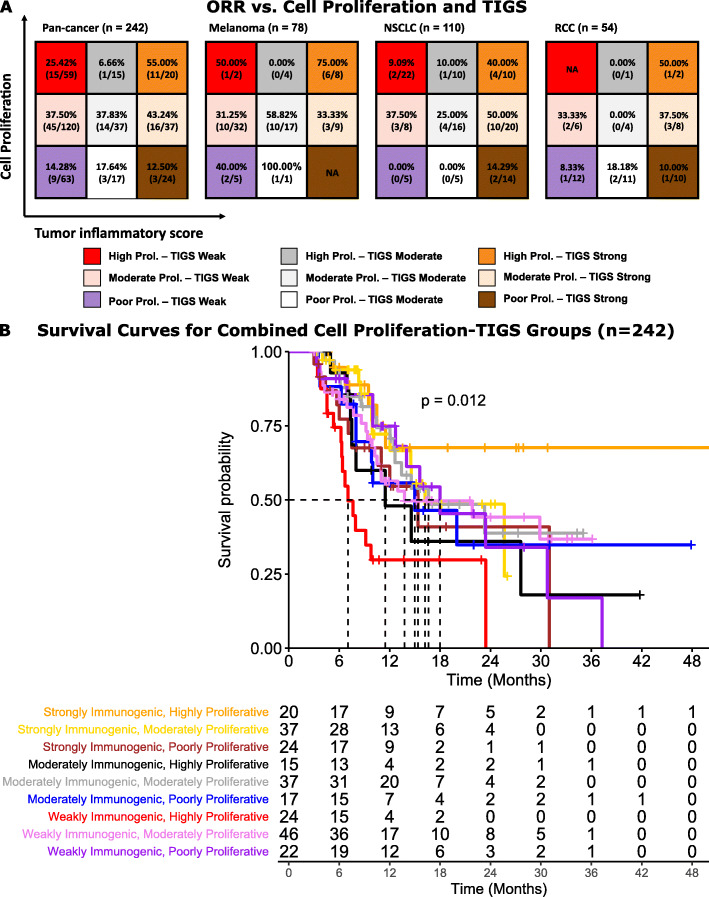
Retrospective cohort combining TIGS and cell proliferation to determine ORR to ICI (A), and survival (B). **A.** Clinical ORR for each subgroup in the retrospective cohort when TIGS is used in conjunction with cell proliferation score classification. **B.** Kaplan Meier survival curves of combined TIGS and cell proliferation status for 242 ICI treated retrospective cohort

In order to gain more comprehensive insight into the TME and its effect on immunotherapy response, an understanding of both immune and neoplastic influences is required. To achieve this, we combined TIGS with a previously published emerging biomarker of cell proliferation [12, 14]. Combining TIGS subgroups with cell proliferation classes of highly, moderately, and poorly proliferative tumors significantly improves objective response separation, where highly proliferative, inflamed tumors [55%] have significantly higher objective response to ICI therapy than poorly proliferative, non-inflamed tumors [14.28%; *p* = 0.0006] [Fig. 4a]. Tumor type-specific analysis were not performed due to small sample sizes within each group.

**Fig. 5:**
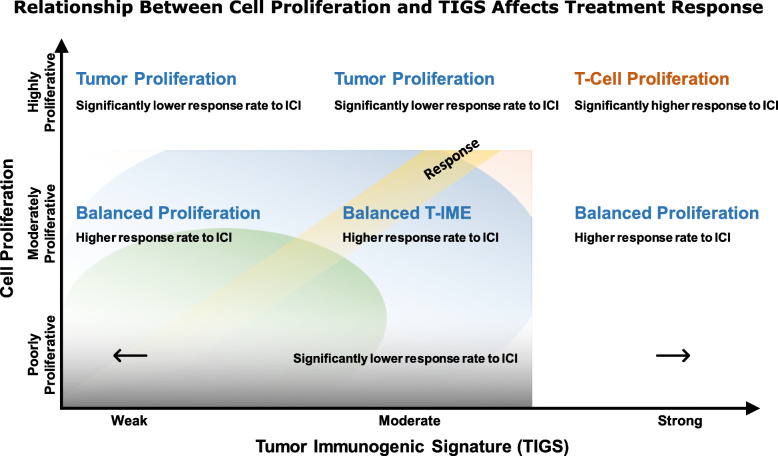
Integrative hypothesis for utility of TIGS and cell proliferation for treatment selection. Hypothesized relationship mechanism by which cell proliferation and tumor immunogenicity affect treatment response

Further evidence demonstrated significant survival differences between different combinations of TIGS and cell proliferation [*p* = 0.012] [Fig. 4b]. Importantly, we noted that strongly inflamed and highly [median = not achieved; *p* = 0.025] or moderately [median = 16.2 months; *p* = 0.025] proliferative tumors had significantly better survival compared to weakly inflamed, highly proliferative tumors [median = 7.03 months] [Tables S6, S7]. Even though this difference was not statistically significant for individual tumor types, median OS was not reached for strongly inflamed and highly proliferative tumors for NSCLC and melanoma [Supplementary Fig. S9, S10, S11]. Multivariate Cox proportional hazard analysis showed no additional effect of age, gender, PD-L1 IHC status, and TMB status on the overall survival [Supplementary Fig. S8]. This data suggests and we hypothesize that both T cell proliferation and tumor cell proliferation contribute to the signal in highly inflamed and highly proliferative tumors, whereas only tumor cell proliferation appears to contribute to the measurement of highly proliferative, weakly inflamed tumors [Fig. 5]. Therefore, combining biomarkers of both neoplastic and immune influences as described could facilitate a more comprehensive understanding of the tumor immune microenvironment and likelihood of response to ICIs.

## Discussion

Even though PD-L1 expression and TMB are among the most utilized biomarkers for ICI treatment decision making [13, 15], the complexity of the antitumor host immune response cannot be fully explained by a single biomarker of immune or neoplastic mechanism. TMB is known to be correlated to response to ICI in multiple disease types however when evaluated for combination therapy there is no reported difference in median TMB for responders versus non-responders [16]. Since TMB does not directly represent the neoantigen load comprised of immunogenic neopeptides, it may only lead to limited understanding of the TME being assessed. Similarly, PD-L1 by IHC was only found to be predictive in 28.9% of cases across 45 FDA drug approvals for ICI across 15 tumor types [17]. This results in the need to investigate multiplex biomarkers, including tumor immunogenic signature, that are more comprehensive in deciphering the state of the tumor immune microenvironments primed for ICI response.

To enable a more comprehensive treatment decision, a robust measurement of the host immune response is required [8]. In this study we show the discovery of comprehensive gene expression-based tumor immunogenic signature (TIGS) that complements both traditional and emerging biomarkers of ICI response in solid tumors. TIGS was derived from a pan-cancer cohort of real-world clinical FFPE tumors to broadly describe immunogenic state of the TME as strongly, moderately and weakly inflamed. The TIGS score was highly correlated to the TIL infiltration pattern observed in the tumor samples and differentiated patients with higher response and improved survival in NSCLC. TIGS score also complemented traditional biomarkers where, as expected PD-L1^+^ tumors that were strongly inflamed had a very high response (45%; 18/40). Interestingly, TIGS was able to identify a subpopulation of PD-L1 negative tumors with a strongly inflamed phenotype with response to ICI up to 29% (12/41). Similarly, TIGS complements TMB where TMB high tumors that are strongly inflamed have ORR of 48% (13/17), but was also able to identify non-TMB high, strongly inflamed cases that have ORR of 31% (17/54) to ICI. Specifically focusing on NSCLC which is the largest population of the discovery cohort, we observe the clinical utility of TIGS in this disease type. After conducting a retrospective analysis of 110 NSCLC samples using the clinically recommended immune checkpoint biomarkers of PD-L1 and TMB by targeted next generation sequencing, we identified a substantial subpopulation of PD-L1-, TMB- patients (24%; *n* = 26) of which 46% presented an inflamed TME as measured by TIGS. These PD-L1-, TMB-, TIGS inflamed patients had ORR of 42% whereas none of the PD-L1-, TMB- and moderately or weakly inflamed tumors responded to ICI [Supplementary Table S8]. As such, we believe our TIGS may serve as a novel method to identify a substantial cohort of NSCLC patients who would benefit from ICI that would not be identified by current clinical protocols.

Next, we combined our TIGS with cell proliferation which is an emerging biomarker for resistance to ICI therapy in NSCLC and RCC [8, 11]. As previously published, moderately proliferative tumors demonstrate significantly higher response to ICI as compared to poorly or highly proliferative tumors regardless of immunogenicity, except in the case of highly inflamed tumors. Highly inflamed and highly proliferative tumors had the highest response rate in the retrospective cohort. This led us to hypothesize that TIGS represents the host immune response and cell proliferation represents the overall proliferative potential of the entire TME. In case of strongly inflamed and highly proliferative tumors, the cell proliferation signal can be attributed to antigen stimulated T cell proliferation as well as neoplastic cell proliferation. This TME is uniquely primed for response to ICI therapy. However, weakly inflamed tumors may not contribute to cell proliferation signal via antigen stimulated T cell proliferation. Therefore, most of the cell proliferation signal may be attributed to neoplastic cell proliferation making the TME resistance to ICI therapy due to lack of underlying host immune response. Combining the TIGS and cell proliferation with traditional biomarkers of PD-L1 and TMB support this merger. Hence, in the retrospective cohort we were able to identify PD-L1-, TMB- patients that had very high response rate for highly proliferative, strongly inflamed tumors (100%; 2/2) and moderately proliferative, strongly inflamed tumors (42%; 5/12) [Supplementary Table S9]. As such, we believe our TIGS in conjunction with traditional and emerging biomarkers of ICI response and resistance may provide comprehensive understanding of the underlying state of immune and neoplastic influences that contribute to the success of failure of ICI therapy.

Even though our work is not based on controlled trial samples, we derived the immunogenic score from a large cohort of real world clinical FFPE samples spanning multiple solid tumor types. One of the major limitations of this work is lack of subgroup sample size to perform sufficiently powered analysis when multiple biomarkers are combined. This led to a pooled analysis of the retrospective cohort by ICI treatment agent. Additionally, the small sample size for the RCC and melanoma retrospective cohort limits the analysis which could be performed on a subgroup level. Due to a lack of frontline checkpoint treatment data along with aforementioned limitations, we believe that further studies are warranted to investigate additional tumor type- and treatment type-specific effects of TIGS alone and in conjunction with other biomarkers in the context of frontline checkpoint inhibition therapy. Additionally, this study could benefit from further investigation into the correlation of immune composition of peripheral blood within the tumor-immune microenvironment and immunotherapy response. On the other hand, this study also does not address the immune and neoplastic influences observed in the non-treated tumors which might bring additional discrimination of prognostic versus predictive abilities of combination of TIGS and cell proliferation. However, we believe this large-scale assessment of a clinical grade cohort will lead to further hypothesis testing of integration of immune and neoplastic signals in the tumor immune microenvironment.

## Conclusions

In summary, we describe a comprehensive tumor immunogenic signature which portrays the underlying host immune response and also mediates the aggregation of primary biomarkers of ICI response (PD-L1 and TMB) along with biomarkers of resistance such as cell proliferation. TIGS alone as well as in combination with these biomarkers can identify patient subpopulations that may be resistance to ICI therapy but more importantly select patients that may have not been identified for response to ICI by traditional clinical biomarkers. The gene expression assay which measures TIGS can be implemented into routine practice to promote drug development efforts, facilitate patient selection for clinical trials and support treatment decision making as part of routine clinical care.

## Supplementary Information

**Additional file 1: Fig. S1.** Gene expression rank calculation workflow. **Fig. S2.** Tumor immunogenic signature discovery workflow. **Fig. S3.** Effects of TIGS category, tumor type, sex, age, TMB status, and PD-L1 IHC on survival in retrospective cohort, as determined by multivariate Cox proportional hazard model analysis. **Fig. S4.** Effects of TIGS category, sex, age, TMB status, and PD-L1 IHC on melanoma survival in retrospective cohort, as determined by multivariate Cox proportional hazard model analysis. **Fig. S5.** Effects of TIGS category, sex, age, TMB status, and PD-L1 IHC on lung cancer (NSCLC) survival in retrospective cohort, as determined by multivariate Cox proportional hazard model analysis. **Fig. S6.** Effects of TIGS category, sex, age, TMB status, and PD-L1 IHC on kidney cancer (RCC) survival in retrospective cohort, as determined by multivariate Cox proportional hazard model analysis. **Fig. S7.** Clinical response rates in the retrospective cohort for each TIGS subgroup when used in combination with TMB and PD-L1 IHC. **Fig. S8.** Effects of TIGS used in combination with cell proliferation category, sex, age, TMB status, and PD-L1 IHC on survival in retrospective cohort, as determined by multivariate Cox proportional hazard model analysis. **Fig. S9.** Retrospective cohort combining TIGS and cell proliferation to determine survival in melanoma. **Fig. S10.** Retrospective cohort combining TIGS and cell proliferation to determine survival in NSCLC. **Fig. S11.** Retrospective cohort combining TIGS and cell proliferation to determine survival in RCC.

**Additional file 2: Table S1.** Discovery cohort histological, PD-L1, TMB and TIGS data. **Table S2.** Panther analysis of gene pathways in TIGS cluster. **Table S3.** Pathway analysis of gene pathways in immune and other neoplasm cluster. **Table S4.** Objective response rates for TIGS groups in retrospective cohort for each disease type. **Table S5.** Aggregate survival data for retrospective cohort. **Table S6.** Median survival for combined TIGS and Cell Proliferation groups. **Table S7.** Pairwise comparison *p*-values for survival of retrospective cohort when grouped by TIGS and cell proliferation. **Table S8.** Objective response rates for a subpopulation of PD-L1 – and TMB low (*n* = 26) of the NSCLC retrospective cohort for three TIGS groups. **Table S9.** Objective response rates for retrospective cohort subdivided by PD-L1 status, TMB status, cell proliferation classification, and TIGS. **Table S10.** Sequencing coverage and quality statistics for discovery cohort. **Table S11.** Sequencing coverage and quality statistics for retrospective cohort.

## Data Availability

The datasets generated and/or analyzed during the current study are not publicly available due to a non-provisional patent filing covering the methods used to analyze such datasets but are available from the corresponding author on reasonable request.

## Abbreviations

TIGS: Tumor immunogenic signature
ICI: Immune checkpoint inhibitor
PD-L1: Programmed death-ligand 1
PD-1: Programmed cell death 1
TMB: Tumor mutational burden
CTLA4: Cytotoxic T-lymphocyte associated protein 4
IHC: Immunohistochemistry
FFPE: Formalin-fixed paraffin-embedded
QC: Quality control
IRB: Institutional review board
RECIST: Response evaluation criteria in solid tumors
OS: Overall survival
ORR: Overall response rate
CR: Complete response
PR: Partial response
SD: Stable disease
PD: Progressive disease
TCGA: The cancer genome atlas
FDA: Food and drug administration
CLIA: Clinical laboratory improvement amendments
CAP: College of American pathologists
HK: Housekeeping
TPS: Tumor proportion score
