# Supplementary material for: Integration of tumor inflammation, cell proliferation, and traditional biomarkers improves prediction of immunotherapy resistance and response
Source: Biomark Res. 2021 Jul 7;9:56. doi: 10.1186/s40364-021-00308-6 (PMC8265007; doi:10.1186/s40364-021-00308-6)
Supplement: Supplementary file 1 — Additional file 1: Fig. S1. Gene expression rank calculation workflow. Fig. S2. Tumor immunogenic signature discovery workflow. Fig. S3. Effects of TIGS category, tumor type, sex, age, TMB status, and PD-L1 IHC on survival in retrospective cohort, as determined by multivariate Cox proportional hazard model analysis. Fig. S4. Effects of TIGS category, sex, age, TMB status, and PD-L1 IHC on melanoma survival in retrospective cohort, as determined by multivariate Cox proportional hazard model analysis. Fig. S5. Effects of TIGS category, sex, age, TMB status, and PD-L1 IHC on lung cancer (NSCLC) survival in retrospective cohort, as determined by multivariate Cox proportional hazard model analysis. Fig. S6. Effects of TIGS category, sex, age, TMB status, and PD-L1 IHC on kidney cancer (RCC) survival in retrospective cohort, as determined by multivariate Cox proportional hazard model analysis. Fig. S7. Clinical response rates in the retrospective cohort for each TIGS subgroup when used in combination with TMB and PD-L1 IHC. Fig. S8. Effects of TIGS used in combination with cell proliferation category, sex, age, TMB status, and PD-L1 IHC on survival in retrospective cohort, as determined by multivariate Cox proportional hazard model analysis. Fig. S9. Retrospective cohort combining TIGS and cell proliferation to determine survival in melanoma. Fig. S10. Retrospective cohort combining TIGS and cell proliferation to determine survival in NSCLC. Fig. S11. Retrospective cohort combining TIGS and cell proliferation to determine survival in RCC. [file 40364_2021_308_MOESM1_ESM.zip › FigS1_04052021.pdf]

Gene Expression Rank Calculation

Gene Expression Rank

	STEP	Description
Ranked	4 Rank	For each gene, gene expression rank is calculated as nRPM percentile against a reference population of 735 tumors. Rank for transcript (t) will calculated as $\text{Rank}_{(t)} = 100 \times \frac{\#\text{ of samples in Reference population} < \text{nRPM}_{(t)}}{735}$
Normalized	3 Normalized Reads Per Million	$\text{nRPM}_{(t)} = \frac{\text{Background subtracted absolute read count}_{(t)}}{\text{Normalization ratio}}$, Where Normalization ratio = $\frac{\text{Background subtracted abs read count of house keeping genes}}{\text{Pre-defined reads per million profile of house keeping genes}}$
Raw	2 Background subtracted Absolute read Count	For each transcript (t), Background subtracted Absolute read Count = $(\text{absolute read counts}(t) - \text{absolute read counts } from\ NTC\ (t))$
Raw	1 Absolute Read Count	RNA-seq absolute reads for each transcript (t) were generated with Torrent Suite's plugin immuneResponseRNA.
