# Supplementary material for: Integration of tumor inflammation, cell proliferation, and traditional biomarkers improves prediction of immunotherapy resistance and response
Source: Biomark Res. 2021 Jul 7;9:56. doi: 10.1186/s40364-021-00308-6 (PMC8265007; doi:10.1186/s40364-021-00308-6)
Supplement: Supplementary file 1 — Additional file 1: Fig. S1. Gene expression rank calculation workflow. Fig. S2. Tumor immunogenic signature discovery workflow. Fig. S3. Effects of TIGS category, tumor type, sex, age, TMB status, and PD-L1 IHC on survival in retrospective cohort, as determined by multivariate Cox proportional hazard model analysis. Fig. S4. Effects of TIGS category, sex, age, TMB status, and PD-L1 IHC on melanoma survival in retrospective cohort, as determined by multivariate Cox proportional hazard model analysis. Fig. S5. Effects of TIGS category, sex, age, TMB status, and PD-L1 IHC on lung cancer (NSCLC) survival in retrospective cohort, as determined by multivariate Cox proportional hazard model analysis. Fig. S6. Effects of TIGS category, sex, age, TMB status, and PD-L1 IHC on kidney cancer (RCC) survival in retrospective cohort, as determined by multivariate Cox proportional hazard model analysis. Fig. S7. Clinical response rates in the retrospective cohort for each TIGS subgroup when used in combination with TMB and PD-L1 IHC. Fig. S8. Effects of TIGS used in combination with cell proliferation category, sex, age, TMB status, and PD-L1 IHC on survival in retrospective cohort, as determined by multivariate Cox proportional hazard model analysis. Fig. S9. Retrospective cohort combining TIGS and cell proliferation to determine survival in melanoma. Fig. S10. Retrospective cohort combining TIGS and cell proliferation to determine survival in NSCLC. Fig. S11. Retrospective cohort combining TIGS and cell proliferation to determine survival in RCC. [file 40364_2021_308_MOESM1_ESM.zip › FigS10_05252021.pdf]

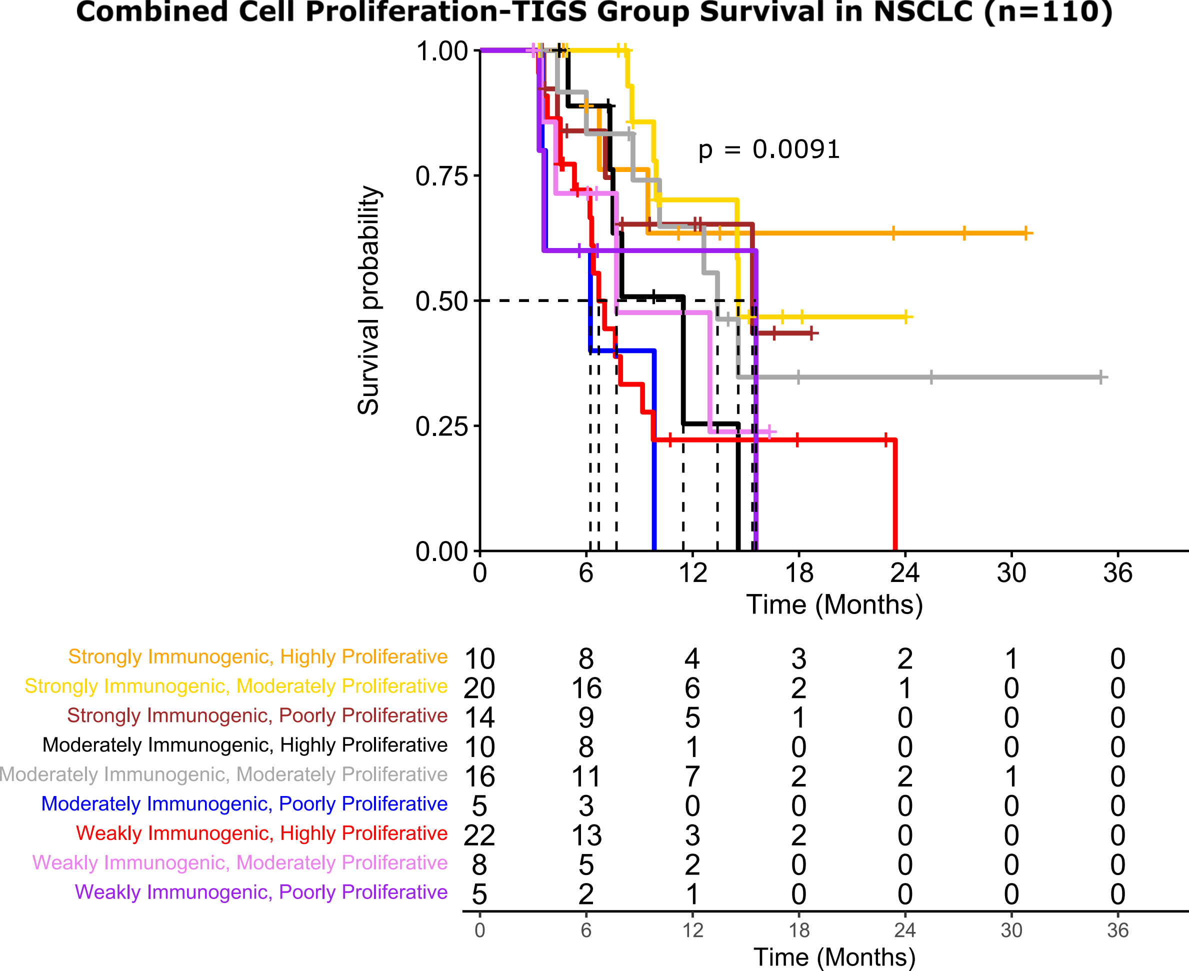

Combined Cell Proliferation-TIGS Group Survival in NSCLC (n=110)
Survival probability
1.00
0.75
0.50
0.25
0.00
p = 0.0091
0 6 12 18 24 30 36
Time (Months)
Strongly Immunogenic, Highly Proliferative 10 8 4 3 2 1 0
Strongly Immunogenic, Moderately Proliferative 20 16 6 2 1 0 0
Strongly Immunogenic, Poorly Proliferative 14 9 5 1 0 0 0
Moderately Immunogenic, Highly Proliferative 10 8 1 0 0 0 0
Moderately Immunogenic, Moderately Proliferative 16 11 7 2 2 1 0
Moderately Immunogenic, Poorly Proliferative 5 3 0 0 0 0 0
Weakly Immunogenic, Highly Proliferative 22 13 3 2 0 0 0
Weakly Immunogenic, Moderately Proliferative 8 5 2 0 0 0 0
Weakly Immunogenic, Poorly Proliferative 5 2 1 0 0 0 0
0 6 12 18 24 30 36
Time (Months)
