# Supplementary material for: Integration of tumor inflammation, cell proliferation, and traditional biomarkers improves prediction of immunotherapy resistance and response
Source: Biomark Res. 2021 Jul 7;9:56. doi: 10.1186/s40364-021-00308-6 (PMC8265007; doi:10.1186/s40364-021-00308-6)
Supplement: Supplementary file 1 — Additional file 1: Fig. S1. Gene expression rank calculation workflow. Fig. S2. Tumor immunogenic signature discovery workflow. Fig. S3. Effects of TIGS category, tumor type, sex, age, TMB status, and PD-L1 IHC on survival in retrospective cohort, as determined by multivariate Cox proportional hazard model analysis. Fig. S4. Effects of TIGS category, sex, age, TMB status, and PD-L1 IHC on melanoma survival in retrospective cohort, as determined by multivariate Cox proportional hazard model analysis. Fig. S5. Effects of TIGS category, sex, age, TMB status, and PD-L1 IHC on lung cancer (NSCLC) survival in retrospective cohort, as determined by multivariate Cox proportional hazard model analysis. Fig. S6. Effects of TIGS category, sex, age, TMB status, and PD-L1 IHC on kidney cancer (RCC) survival in retrospective cohort, as determined by multivariate Cox proportional hazard model analysis. Fig. S7. Clinical response rates in the retrospective cohort for each TIGS subgroup when used in combination with TMB and PD-L1 IHC. Fig. S8. Effects of TIGS used in combination with cell proliferation category, sex, age, TMB status, and PD-L1 IHC on survival in retrospective cohort, as determined by multivariate Cox proportional hazard model analysis. Fig. S9. Retrospective cohort combining TIGS and cell proliferation to determine survival in melanoma. Fig. S10. Retrospective cohort combining TIGS and cell proliferation to determine survival in NSCLC. Fig. S11. Retrospective cohort combining TIGS and cell proliferation to determine survival in RCC. [file 40364_2021_308_MOESM1_ESM.zip › FigS2_04052021.pdf]

Tumor Immunogenic Signature Discovery

Phase 1

Visualization

Hierarchical Clustering

We performed hierarchical clustering of 395 transcript ranks for 1323 samples using Pearson's correlation as measure of distance to visualize broad clusters to further inform k means clustering (Data not shown).

We observed three broad clusters of patients and genes.

Phase 2

K-means Clustering

Unsupervised Clustering

Based on visualizing three broad samples and gene clusters in phase 1, k was set to 3 for samples as well as gene clusters and repeated 100 times until the clusters were stable (Fig 1A).

Phase 3

Pathway Analysis

Pathway Enrichment

Next, we performed Panther pathway enrichment analysis all three gene clusters and identified the 161 gene cluster as immunogenic cluster as it was overrepresented by T and B cell activation pathways as well as IFNg pathway (Table S1).

Phase 4

Tumor Immunogenic Score

Thresholds

Tumor Immunogenic Score was calculated as mean expression rank of 161 genes. To classify this immunogenic score, we estimated thresholds based on the clusters in Phase 2 as Median ± 2SD for each k means cluster. This led to the following groups:

Strong = TIGS ≥ 62

Moderate = TIGS > 43 & < 62

Weak = TIGS ≤ 43
