# Supplementary material for: Integration of tumor inflammation, cell proliferation, and traditional biomarkers improves prediction of immunotherapy resistance and response
Source: Biomark Res. 2021 Jul 7;9:56. doi: 10.1186/s40364-021-00308-6 (PMC8265007; doi:10.1186/s40364-021-00308-6)

Multivariate Cox Proportional Hazard Analysis of Covariable Effects on Kidney Cancer (RCC) Survival in Retrospective Cohort

Survival: HR (95% CI, p-value)		
TIGS	Strong	-
	Moderate	0.98 (0.38-2.51, p=0.965)
	Weak	0.62 (0.25-1.50, p=0.287)
Sex	F	-
	M	0.70 (0.27-1.81, p=0.467)
Age_Group	(27.9,48]	-
	(48,52]	2.02 (0.66-6.17, p=0.220)
	(52,56.3]	0.65 (0.08-5.15, p=0.687)
	(56.3,61]	1.45 (0.55-3.77, p=0.451)
	(61,64]	0.47 (0.06-3.51, p=0.461)
	(64,67]	1.66 (0.48-5.70, p=0.424)
	(67,70]	0.51 (0.09-2.74, p=0.431)
	(70,72]	20.89 (2.17-200.89, p=0.008)
	(72,76.9]	2.49 (0.56-11.08, p=0.230)
	(76.9,93.1]	0.00 (0.00-Inf, p=0.994)
TMB_Status	TMB High	-
	Non-TMB High	0.87 (0.00-Inf, p=1.000)
PDL1_IHC	Positive	-
	Negative	0.83 (0.30-2.30, p=0.723)

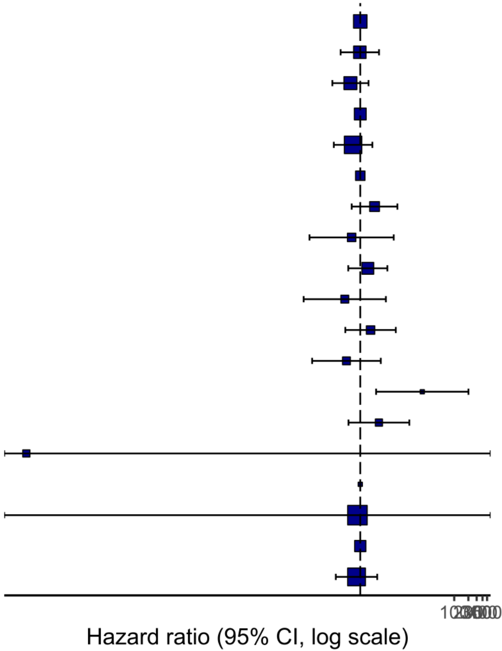

Supplement: Supplementary file 1 — Additional file 1: Fig. S1. Gene expression rank calculation workflow. Fig. S2. Tumor immunogenic signature discovery workflow. Fig. S3. Effects of TIGS category, tumor type, sex, age, TMB status, and PD-L1 IHC on survival in retrospective cohort, as determined by multivariate Cox proportional hazard model analysis. Fig. S4. Effects of TIGS category, sex, age, TMB status, and PD-L1 IHC on melanoma survival in retrospective cohort, as determined by multivariate Cox proportional hazard model analysis. Fig. S5. Effects of TIGS category, sex, age, TMB status, and PD-L1 IHC on lung cancer (NSCLC) survival in retrospective cohort, as determined by multivariate Cox proportional hazard model analysis. Fig. S6. Effects of TIGS category, sex, age, TMB status, and PD-L1 IHC on kidney cancer (RCC) survival in retrospective cohort, as determined by multivariate Cox proportional hazard model analysis. Fig. S7. Clinical response rates in the retrospective cohort for each TIGS subgroup when used in combination with TMB and PD-L1 IHC. Fig. S8. Effects of TIGS used in combination with cell proliferation category, sex, age, TMB status, and PD-L1 IHC on survival in retrospective cohort, as determined by multivariate Cox proportional hazard model analysis. Fig. S9. Retrospective cohort combining TIGS and cell proliferation to determine survival in melanoma. Fig. S10. Retrospective cohort combining TIGS and cell proliferation to determine survival in NSCLC. Fig. S11. Retrospective cohort combining TIGS and cell proliferation to determine survival in RCC. [file 40364_2021_308_MOESM1_ESM.zip › FigS6_04052021.pdf]
